# Physiological Responses and Yield of Wheat Plants in Zinc-Mediated Alleviation of Drought Stress

**DOI:** 10.3389/fpls.2017.00860

**Published:** 2017-05-24

**Authors:** Dongyun Ma, Dexiang Sun, Chenyang Wang, Huina Ding, Haixia Qin, Junfeng Hou, Xin Huang, Yingxin Xie, Tiancai Guo

**Affiliations:** ^1^Agronomy College/National Engineering Research Center for Wheat, Henan Agricultural UniversityZhengzhou, China; ^2^The Collaborative Innovation Center of Henan Food Crops, Henan Agricultural UniversityZhengzhou, China; ^3^The National Key Laboratory of Wheat and Maize Crop Science, Henan Agricultural UniversityZhengzhou, China

**Keywords:** wheat, physiological responses, grain yield, zinc fertilizer, drought

## Abstract

To evaluate the physiological responses of wheat to zinc (Zn) fertilizer application under drought stress, pot, and field experiments were conducted on wheat plants grown under different soil moistures and treated with soil and foliar Zn applications. Photosynthetic characteristics, antioxidant content, Zn element concentration, and the transcription level of genes involved in antioxidant biosynthesis were analyzed. Zn application increased SPAD and *Fv/Fm* of wheat flag leaves, while decreased lipid peroxidation levels and H_2_O_2_ content. Zn application increased the antioxidant content (ascorbate, reduced glutathione, total phenolic, and total flavonoid) of wheat flag leaves, and enhanced the relative expression levels of two antioxidant enzyme genes, four ascorbate–glutathione cycle genes, and two flavonoid biosynthesis pathway genes under drought stress. Soil Zn application increased grain yield and Zn concentration by 10.5 and 15.8%, 22.6 and 9.7%, and 28.2 and 32.8% under adequate water supply, moderate drought, and severe drought, respectively. Furthermore, foliar application of Zn in the field increased grain yield and grain Zn concentration under both adequate water supply and rain-fed conditions. Zn plays a role in alleviating wheat plant drought stress by Zn-mediated increase in photosynthesis pigment and active oxygen scavenging substances, and reduction in lipid peroxidation. Furthermore, Zn fertilizer could regulate multiple antioxidant defense systems at the transcriptional level in response to drought.

## Introduction

Wheat is a major staple food crop in the world. Increasing grain yield and improving quality are of great importance for the increasing human population (Curtis and Halford, 2014). However, drought is considered a key environmental stress that adversely affects wheat growth and yield (Xue et al., 2014). Drought results in increased reactive oxygen species (ROS), which can damage cellular components (Iturbe-Ormaetxe et al., 1998). To counteract the toxicity of ROS, plants have developed complex enzymatic and non-enzymatic antioxidant defense systems. Superoxide dismutase (SOD) scavenges superoxide radicals, while catalases (CAT) the transformation of H_2_O_2_ to H_2_O (Gill and Tuteja, 2010). These ROS scavenging enzymes play an important role in the first line of defense. The ascorbate–glutathione (ASC–GSH) cycle in plants plays an important role in detoxifying ROS (Liang et al., 2003). The cycle involves the antioxidant metabolites: ASC and GSH, and the enzymes linking these metabolites. Glutathione reductase (GR), monodehydroascorbate reductase (MDHAR), dehydroascorbate reductase (DHAR), and ascorbate peroxidase (APX) are important enzymes involved in the ASC–GSH cycle (Li et al., 2013). Recently, it has been suggested that flavonoids could inhibit the generation of ROS, and reduce levels of ROS once formed in response to environmental stress (Agati and Tattini, 2010). Some studies showed that low molecular weight antioxidants like ASC and GSH, as well as flavonoids, play an important role in defense against ROS induced by abiotic stress (Liang et al., 2003; Ma et al., 2014).

Zinc (Zn) is one of the important elements for plant growth and plays an important role as a metal component, or as a functional, structural, or regulatory cofactor of many enzymes (Marschner, 1995). Results showed that foliar application of Zn increased the number of wheat grains per spike, and increased the seed yield and oil percentage of corn (Soleimani, 2006; Tabatabai et al., 2015). Moreover, application of Zn fertilizers is one of the important approaches of biofortification of valuable crops for human health. Cakmak et al. (2010) reported that foliar application of Zn significantly increased Zn concentration in whole grain, and the increase was pronounced when Zn was sprayed at the late growth stage (e.g., milk and dough). Hussain et al. (2012) found that soil Zn application increased grain yield (29%), whole-grain Zn concentration (95%), and whole-grain estimated Zn bioavailability (74%).

Zn plays an important role in lowering ROS generation and defending cells against ROS attack, while Zn deficiency can induce higher levels of ROS causing plant damage (Cakmak, 2000). Bagci et al. (2007) found soil Zn deficiency stress became more pronounced when wheat plants were drought stressed; the effect of irrigation on grain yield was maximized when Zn was adequately supplied. Tabatabai et al. (2015) reported that zinc sulfate played a more important role in stomata regulation and ion balance in plant systems to reduce the tension of drought. Foliar application of Zn resulted in a significant increase in SOD, POD, and CAT activity in response to drought stress (Yavas and Unay, 2016). Salt and cadmium stress was alleviated by Zn application (Hassan et al., 2005; Tavallali et al., 2010). Karim et al. (2012) reported that foliar application of Zn did not affect wheat grain yield in the absence of drought, but increased grain yield (15%) and raised grain Zn concentration under drought. Sultana et al. (2016) found that foliar Zn application counteracted the adverse effect of water deficit on wheat yield. However, Zn fertilizer application was recommended for maize plants irrigated or supplied with adequate water (Wang and Jin, 2007).

Although, it is well-documented in previous reports that the application of Zn can increase seed yield and improve the resistance or tolerance of plants to environmental stress, the underlying molecular mechanisms of this effect remain poorly understood. In this study, wheat plants were grown in pots under three different soil moisture conditions including adequate water (AW) supply, moderate drought (MD), and severe drought (SD) and treated with two Zn application levels. Additionally, foliar applications of Zn were conducted in the field under well-watered and rain-fed environments. The objectives of this study were to (1) investigate the response of wheat growth to Zn application under variable soil water supply; (2) determine the effect of Zn application on ROS damage caused by drought stress; (3) investigate the effects of Zn application on wheat grown under drought conditions at the transcriptional level.

## Materials and methods

### Pot experiment I

A preliminary test was conducted during the cropping year 2011/2012 in Henan Agricultural University Experimental Station. Seeds of wheat (*Triticum aestivum* L.) cv. Yumai 49–198 were surface sterilized with 1% sodium hypochlorite for 10 min, allowed to germinate for 24 h, and then sown in plastic pots (diameter 25 cm, height 25 cm) filled with 15 kg of soil. The soil was sieved (1.0 cm) prior to use. The soil was a loamy fluvoaquic with organic matter content of 17.35 g kg^−1^ (0–20 cm), available phosphorus 18.07 mg kg^−1^, available potassium 240.16 mg kg^−1^, DTPA-extractable Zn 1.15 mg kg^−1^, pH 7.75. Before sowing, the soil was fertilized with N/P/K (3.0/2.0/2.0 g pot^−1^) and mixed sufficiently. Two levels of Zn, 0 (Zn_0_), and 14 mg kg^−1^ (Zn_14_) soil, were applied to soil as solutions of ZnSO_4_.7H_2_O before sowing. After emergence, the seedlings were thinned to 10 plants per pot and watered every 3–4 days to maintain a well-watered level until the heading stage. Thereafter, the plants were watered every 1–2 days to maintain 75 ± 5% relative water content in the adequate water supply (AW), 55 ± 5% relative water content in the moderate drought treatments (MD). The amount of water was calculated according to the soil water content and was added using a measuring cylinder. Soil water content was monitored by soil moisture equipment (TDR300, Spectrum, USA) coupled to a 20 cm length probe. Water treatments were assigned to main plots, and Zn fertilizer treatments were applied to the subplots. Each treatment was replicated three times and each replicate comprised four pots. The drought stress lasted for 25 days. At maturity, the number of spikes, the number of kernels per spike and thousand kernel weight (TKW) were measured. Grain Zn concentration was also tested.

### Pot experiment II

The experiment was conducted during the year 2013/2014, in Henan Agricultural University Experimental Station. The soil was a loamy fluvoaquic with organic matter content of 17.47 g kg^−1^ (0–20 cm), available phosphorus 18.83 mg kg^−1^, available potassium 252.56 mg kg^−1^, DTPA-extractable Zn 1.10 mg kg^−1^, pH 7.79. The treatment of soil and seeds was the same as for experiment I. From the heading stage, the plants were watered to maintain three water conditions: AW, MD, and 35 ± 5% relative water content in the severe drought treatments (SD). Water treatments were assigned to main plots, and Zn fertilizer treatments were applied to the subplots. Each treatment was replicated three times and each replicate comprised six pots. The drought stress lasted for 25 days. The flag leaves were collected at ~09:00, 10 and 20 days after stress (DAS) and immediately frozen in liquid N_2_ prior to analysis. The malondialdehyde (MDA) and hydrogen peroxide (H_2_O_2_) content, ascorbate, glutathione, total phenolic content, and total flavonoids content, and the relative expression level of genes involved in antioxidant defense in flag leaves were analyzed. Photosynthetic characteristics (SPAD and chlorophyll fluorescence parameter *Fv/Fm*) were test at 10 and 20 DAS. At maturity, the number of spikes, the number of kernels per spike, and thousand kernel weight (TKW) were measured. Finally, grain Zn concentration was tested.

#### Field experiment

A field experiment was carried out in Henan Agricultural University Experimental Station during 2013/2014 cropping year. The experiment involved a split plot design for winter wheat (*T. aestivum* L.) cv. Yumai 49–198. Irrigation treatments were assigned to main plots, and foliar nutrient treatments were applied to the subplots. The soil nutrient content was the same as experiment II. The soil bulk density 1.28 g cm^−3^, field capacity 21.9%, sand 32.3%, silt 57.7%, and clay 10.0%. Before sowing, each plot received the same amount of N (120 kg N ha^−1^), P (P_2_O_5_, 120 kg ha^−1^), and K (K_2_O, 120 kg ha^−1^). Nitrogen fertilization was applied with two splits: 50% broadcast before sowing and 50% top-dressed at the elongation stage. The area of the sub-plot was 18 m^2^. All plots were treated with the same cultivation management until the jointing stage. After the jointing stage, two irrigation treatments were applied to the main plots: adequate water (AW) and rain-fed (RF). The irrigated plots were watered by a movable sprinkling system with irrigating quota 750 m^3^ ha^−1^ each time and the amount of water was calculated using a water meter. Two treatments were applied as foliar sprays with a mini-directed jet sprayer in each treatment including Zn_0_ (deionized water) and Zn_0.4_ (0.4% ZnSO_4_.7H_2_O). Foliar sprays were applied four times, once every 10 d in the flagging to grain filling stages. The volume of foliar spray was 0.08 L m^−2^. Photosynthetic characteristics (SPAD and *Fv/Fm*) were tested at 10 and 20 days after flowering (DAF). Planting date was 12 October 2013 and weeding was performed manually. Plants in 8 m^2^ of each plot were harvested by hand during the first week of June. Grain yields were determined by weighing the harvested seed, which had been dried to a standard 13% moisture content.

### Determination of SPAD value and chlorophyll fluorescence parameter *Fv/Fm*

The SPAD values of six flag leaves per pot were measured with SPAD-501 chlorophyll meter (Minolta, Japan). The chlorophyll fluorescence parameter *Fv/Fm* was measured with FMS-2 portable fluorometer (Hansatech, Brish). Before each measurement, leaves were dark-adapted for at least 20 min with leaf-clips.

### Determination of malondialdehyde (MDA) and hydrogen peroxide (H_2_O_2_)

MDA was measured using the thiobarbituric acid method. Leaf samples (0.5 g) were homogenized in 4.0 mL of 10 % trichloroacetic acid (TCA) and centrifuged at 10,000 × g for 10 min at 4°C. The supernatant was assayed for MDA following the method of Sairam and Srivastava (2001). Leaf hydrogen peroxide content was measured as described by Velikova et al. (2000). Leaf material (0.1 g) was homogenized on ice in 0.1% (w/v) TCA. The homogenate was centrifuged at 10,000 × g for 15 min at 4°C, and a 0.5 mL sample of the supernatant was combined with 0.5 mL of 10 mM potassium phosphate buffer (pH 7.0) and 1 mL of 1 M KI. The absorbance of the assay mixture was read at 390 nm and the content of H_2_O_2_ was calculated based on a standard curve of known concentrations of H_2_O_2_.

### Determination of ascorbate, glutathione, total phenolic content, and total flavonoids content

The contents of ascorbate (ASC) were measured according to Foyer et al. (1983). Briefly, a 200 mg sample of leaves was ground in liquid nitrogen and 1 mL 2.5 M perchloric acid. The crude extract was centrifuged at 4°C for 10 min at 10,000 × g, and the supernatant was neutralized with saturated K_2_CO_3_. The neutralized extract was added to 3 mL of a reaction mixture containing 20 mM dithiothreitol (DTT) in 50 mM Hepes–KOH (pH 7.0). After incubation for 10 min at 25°C in a water bath, 100 μL of 0.5 M N-ethylmaleimide was added to remove DTT. The reaction was started with the addition of 5 U of ascorbate oxidase. The absorbance at 265 nm was measured with a spectrophotometer.

Reduced glutathione (GSH) was determined according to Griffith (1980). Wheat leaves (200 mg) were extracted in 1 mL of an ice-cold mix of 0.1 M HCl and 0.1 mM EDTA. The neutralized supernatant was used for the assay of GSH and oxidized glutathione (GSSG). The difference between total glutathione and GSSG content is presented as the GSH content.

Total phenolic content (TPC) and total flavonoid content (TFC) were measured according to Shen et al. (2009) with minor modifications. Extracts (0.3 mL) were mixed with 2 mL Folin–Ciocalteu reagent, and the reaction was neutralized with the addition of 1.6 mL saturated sodium carbonate (75 g L^−1^). The mixture was incubated at ambient temperature for 2 h. The absorbance at 765 nm was measured with a spectrophotometer. Aliquots of 0.5 mL of appropriately diluted extracts were pipetted into 15 mL polypropylene conical tubes containing 2 mL double distilled H_2_O and mixed with 0.15 mL 5% NaNO_2_. After 5 min, 0.15 mL 10% AlCl_3_.6H_2_O solution was added, and the mixture was allowed to stand for an additional 5 min, followed by the addition of 1 mL 1 M NaOH. The reaction solution was mixed well and incubated for 15 min, and the absorbance was then determined at 415 nm.

### Determination of Zn concentration in grain

The method described by Shi et al. (2010) was used to measure the nutrient concentrations in samples. All samples of grain were digested by using a HNO_3_-H_2_O_2_ mixture in a microwave-accelerated reaction system (CEM, USA), and nutrient concentrations (Zn) were measured by inductively coupled plasma atomic emission spectrometry (ICP-AES, OPTIMA 3300 DV).

### RNA extraction and real-time PCR

Total RNA was isolated from leaf samples using TriZol Reagent (Invitrogen) according to the manufacturer's instructions. Three PCRs were performed per sample to obtain the average expression level and the standard. Gene expression analysis was performed using SYBR Premix ExTaq (TaKaRa Biotechnology [Dalian] Co., Ltd.), and the experiments were performed according to the manufacturer's instructions. The primer sets used for amplification antioxidant enzyme genes (*SOD* and *CAT*), four enzymes genes involved in the ASC-GSH cycles including *GR, MDHAR, DHAR*, and *APX*, and genes for flavonoid biosynthesis enzymes including *PAL* and *CHS*, as well as reference sequence numbers, are listed in Table 1.

**Table 1:** **Names and sequences of oligonucleotide primers used for gene amplification**.

**Gene**	**Primer**	**Sequence (5′–3′)**	**Reference sequence**
*SOD*	F	AAGCACCACGCCACCTAC	TAU72212
	R	TGGGCTTGAGGTTCTTCC	
*APX*	F	GCCCTCTTGTGGAGAAATA	AJ006358
	R	CTGACAGCGTTCAAGGTAT	
*CAT*	F	GTTGGACGGATGGTACTGA	X94352
	R	AAGACGGTGCCTTTGGGT	
*GR*	F	ATGAATACTCCCGTACATCAGT	AY364467
	R	TTTGTTACATCACCCACAGC	
*DHAR*	F	GTGCCTGTGTATAACGGTG	AY074784
	R	ACAAGTGATGGAGTTGGGT	
*MDHAR*	F	AGAAGTTTACGCCCTTCGGC	AK371371
	R	TTGGAATGTCATCGCCATC	
*PAL*	F	CACCACCCTGGACAGATTG	AY005474
	R	TGAGGCGAAGTGCGGAG	
*CHS*	F	ATGGCGGCTACAATGACG	AY286095
	R	TGTGCTCGCTCTTGGTGA	

### Data analysis

Data were analyzed and evaluated by Statistical Program for Social Science (SPSS) software with UNIANOVA under general linear model (GLM). Water treatment (two levels in the field experiment and pot experiment I, and three levels in the pot experiment II) and Zn fertilizer application (two treatments) were all set as fixed factors, and block was set as a random factor. A customization (C) model was selected to analyze water treatment (W), Zn fertilizer (Zn), and the interaction between water and Zn fertilizer (W × Zn). If the interaction was significant, the simple effect analysis was carried out. To further investigate the effect of Zn fertilizer under different soil water conditions, simple effect was analyzed by adding EMMEANS = TABLES(water^*^Zn) COMPARE(Zn)ADJ(LSD) to UNIANOVA. Differences between each Zn fertilizer treatment were evaluated using Fisher's least significant difference (LSD) test; *p* < 0.05 was considered statistically significant.

## Results

### Effect of Zn application on SPAD and *Fv/Fm* of wheat flag leaves

As shown in Table 2 (pot experiment), analysis of variance (ANOVA) indicated W and Zn fertilizer had a significant effect on SPAD both at 10 and 20 DAS, while no significant interaction was observed. The SPAD value decreased in the flag leaves of wheat plants grown under drought stress. Compared with the AW treatment at 20 DAS, MD, and SD treatment decreased the SPAD value by 6.5 and 37.3%, respectively. Zn application significantly increased SPAD value regardless of the water treatment at 20 DAS. Compared with the SPAD value in the leaves of wheat not treated with Zn, the Zn_14_ treated plants had a higher SPAD value by an average of 10.5% at 20 DAS. W treatment, Zn fertilizer, and W × Zn were all significant for *Fv*/*Fm* at 20 DAS. Compared with the AW treatment, drought stress significantly decreased *Fv/Fm* by an average of 5.6% at 20 DAS. Zn application significantly increased *Fv/Fm* levels regardless of the irrigation treatment. Compared with the corresponding Zn_0_ treatment, Zn_14_ application significantly increased *Fv/Fm* levels by 3.8, 8.1, and 5.4% under AW, MD, and SD treatment at 20 DAS, respectively.

**Table 2:** **Effect of Zn fertilizer application on SPAD and ***Fv/Fm*** of wheat flag leaves under different water conditions^a^^,^^b^^,^^c^^,^^d^^,^^e^**.

**Treatments**		**SPAD**		***Fv/Fm***	
**Pot experiment II**		**10 DAS**	**20DAS**	**10DAS**	**20DAS**
AW	Zn_0_	54.8 ± 0.1a	25.4 ± 0.5b	0.81 ± 0.03b	0.79 ± 0.02b
	Zn_14_	56.0 ± 0.8a	27.2 ± 0.9a	0.84 ± 0.01a	0.82 ± 0.01a
	Mean	55.42A	26.3A	0.83A	0.81A
MD	Zn_0_	53.4 ± 0.5b	23.2 ± 0.2b	0.79 ± 0.02a	0.74 ± 0.02b
	Zn_14_	55.4 ± 0.2a	26.0 ± 0.7a	0.83 ± 0.04a	0.80 ± 0.03a
	Mean	54.4B	24.6B	0.81A	0.77B
SD	Zn_0_	52.6 ± 0.3a	15.4 ± 0.4b	0.78 ± 0.02a	0.74 ± 0.02b
	Zn_14_	53.1 ± 0.7a	17.5 ± 0.8a	0.83 ± 0.03a	0.78 ± 0.02a
	Mean	52.8C	16.5C	0.81A	0.76B
	W	^**^	^**^	ns	^*^
	Zn	^**^	^**^	^**^	^**^
	W × Zn	ns	ns	ns	^*^
**Field experiment**		**10 DAF**	**20DAF**	**10DAF**	**20DAF**
AW	Zn_0_	54.9 ± 0.4b	42.6 ± 0.2b	0.83 ± 0.01a	0.84 ± 0.01a
	Zn_0_._4_	55.9 ± 0.3a	46.6 ± 0.6a	0.84 ± 0.01a	0.85 ± 0.01a
	Mean	55.4A	44.6A	0.84A	0.85A
RF	Zn_0_	51.5 ± 0.5b	25.2 ± 0.1b	0.83 ± 0.01a	0.80 ± 0.01b
	Zn_0_._4_	56.8 ± 1.2a	36.9 ± 2.5a	0.84 ± 0.01a	0.83 ± 0.02a
	Mean	54.2B	31.1B	0.84A	0.82B
	W	^*^	^**^	ns	^**^
	Zn	^**^	^**^	ns	^**^
	W × Zn	^**^	^**^	ns	^**^

a *Values expressed as mean ± standard deviation*.

b *Within an irrigation regime and column, mean values followed by different lowercase letters are significantly different (p < 0.05)*.

c *Within a column, mean values for irrigation regimes followed by different uppercase letters are significantly different (p < 0.05)*.

d* and ** *stand for significant different at p < 0.05 and p < 0.01), respectively; ns stand for no significant different*.

e *W and Zn stand for water conditions and Zn fertilizer treatment, respectively*.

For the field experiment (Table 2), irrigation, Zn fertilizer, and W × Zn interaction had significant effects on SPAD values. Compared with AW treatment, SPAD value in the flag leaves of wheat plant under RF significantly decreased. However, foliar spray Zn significantly increased SPAD values both under AW and RF treatment, compared with the corresponding Zn_0_ treatment. For *Fv/Fm* value, irrigation, Zn fertilizer, and W × Zn were all significant 20 DAF. Compared with AW treatment at 20 DAF, RF decreased the *Fv/Fm* value by 3.5%. Foliar Zn application significantly increased *Fv/Fm* value by 3.8% under RF treatment, compared with corresponding Zn_0_ application.

### Zn decreased MDA and H_2_O_2_ content under drought stress

**Figure 1:**
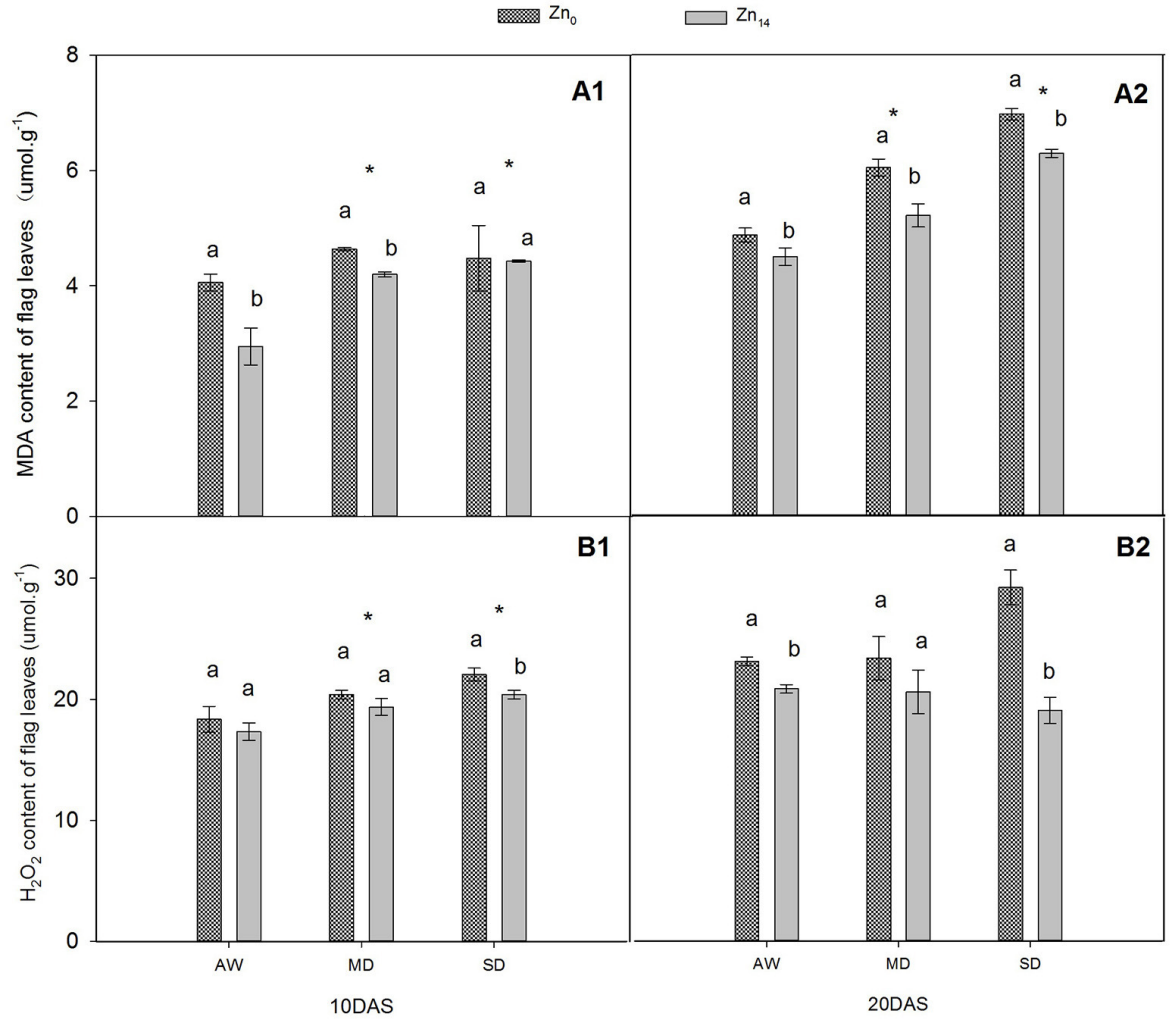
**Effect of Zn application on MDA and H_**2**_O_**2**_ contents of wheat flag leaves under different water conditions**. Vertical bars indicate the standard deviation of the mean; DAS, days after initiation of stress conditions; AW, adequate water; MD, moderate drought; SO, severe drought. Different lower case letters above the columns in the same test period indicate significant differences by LSD method (*p* < 0.05); ^*^above the columns at MD and SD indicate significant differences between drought stress and AW. **(A1, A2)** MDA at 10 DAS and 20 DAS, respectively; **(B1, B2)** H_2_O_2_ at 10 DAS and 20 DAS, respectively.

W treatment and Zn application significantly affected MDA content at 10 and 20 DAS (Figures 1A1,A2). Compared with AW treatment at 20 DAS, MD, and SD stress increased the average MDA content of plant by 20.3 and 41.9%, respectively. However, Zn_14_ application significantly decreased the MDA content in wheat leaves at 20 DAS regardless of soil water conditions. W treatment and Zn significantly affected H_2_O_2_ content 10 DAS (Figure 1B1), while Zn fertilizer and W × Zn interaction were significant at 20 DAS (Figure 1B2). Compared with AW treatment, drought stress increased the average content of H_2_O_2_ by 18.6% at 10 DAS. For SD-treated plants, Zn_14_ application decreased H_2_O_2_ content by 34.7% at 20 DAS, compared with Zn_0_ application.

### Response of antioxidant content of wheat flag leaves to Zn application and drought

**Figure 2:**
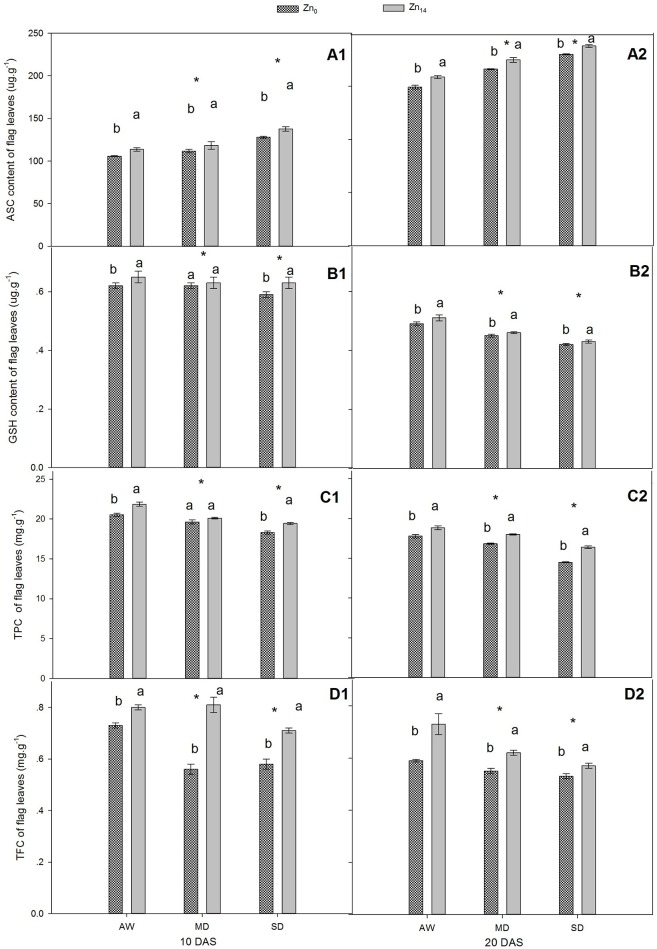
**Effect of Zn application on ASC, GSH, TPC, and TFC content of wheat flag leaves under different water conditions**. Vertical bars indicate the standard deviation of the mean; DAS, days after initiation of stress conditions; AW, adequate water; MD, moderate drought; SO, severe drought; Different lower case letters above the columns in the same test period indicate significant differences by LSD method (*p* < 0.05); ^*^above the columns at MD and SD indicate significant different between drought stress and AW. **(A1, B1, C1, D1)** ASC, GSH, TPC and TFC at 10 DAS, respectively. **(A2, B2, C2, D2)** ASC, GSH, TPC and TFC at 20 DAS, respectively.

As shown in Figures 2A1,A2,B1,B2, W treatment and Zn application all significantly influenced the content of ASC and GSH at 10 and 20 DAS, while a significant interaction was only observed for GSH at 10 DAS. Compared with the well-watered plants, the ASC content increased and GSH content decreased in response to drought stress. Zn_14_ application significantly increased the average ASC content by 11.2 and 9.9% at 10 and 20 DAS, respectively, compared with corresponding Zn_0_ treatment (Figures 2A1,A2). Additionally, GSH content of wheat flag leaves with Zn_14_ application under AW and SD increased by 4.8 and 6.8% at 10 DAS, respectively, compared with Zn_0_ treatment under the same water condition (Figure 2B1).

For TPC and TFC, both W and Zn fertilizer treatment contributed significantly to plant variation at 10 and 20 DAS (Figures 2C1,C2,D1,D2). A significant W × Zn interaction was only observed for TPC at 20 DAS, and TFC at 10 DAS. Compared with corresponding AW treatment, drought stress decreased the TPC content of wheat flag leaves by an average of 8.6 and 10.2% at 10 and 20 DAS, respectively (Figures 2C1,C2). For TFC, the decrease was 13.1 and 14.0% at 10 and 20 DAS, respectively, in comparison with the corresponding value under AW treatment (Figures 2D1,D2). Zn_14_ application significantly increased TPC by an average of 5.0% at 10 DAS, compared with Zn_0_ treatment. At 20 DAS, TPC in wheat flag leaves with Zn_14_ application increased by 5.8, 7.0, and 13.3% under AW, MD, and SD, respectively, compared with corresponding Zn_0_ treatment. Similarly, an increase in TFC at 10 DAS under AW, MD, and SD was 9.5, 44.6, and 22.4%, respectively.

### Response of antioxidant defense to drought stress at transcriptional level

**Figure 3:**
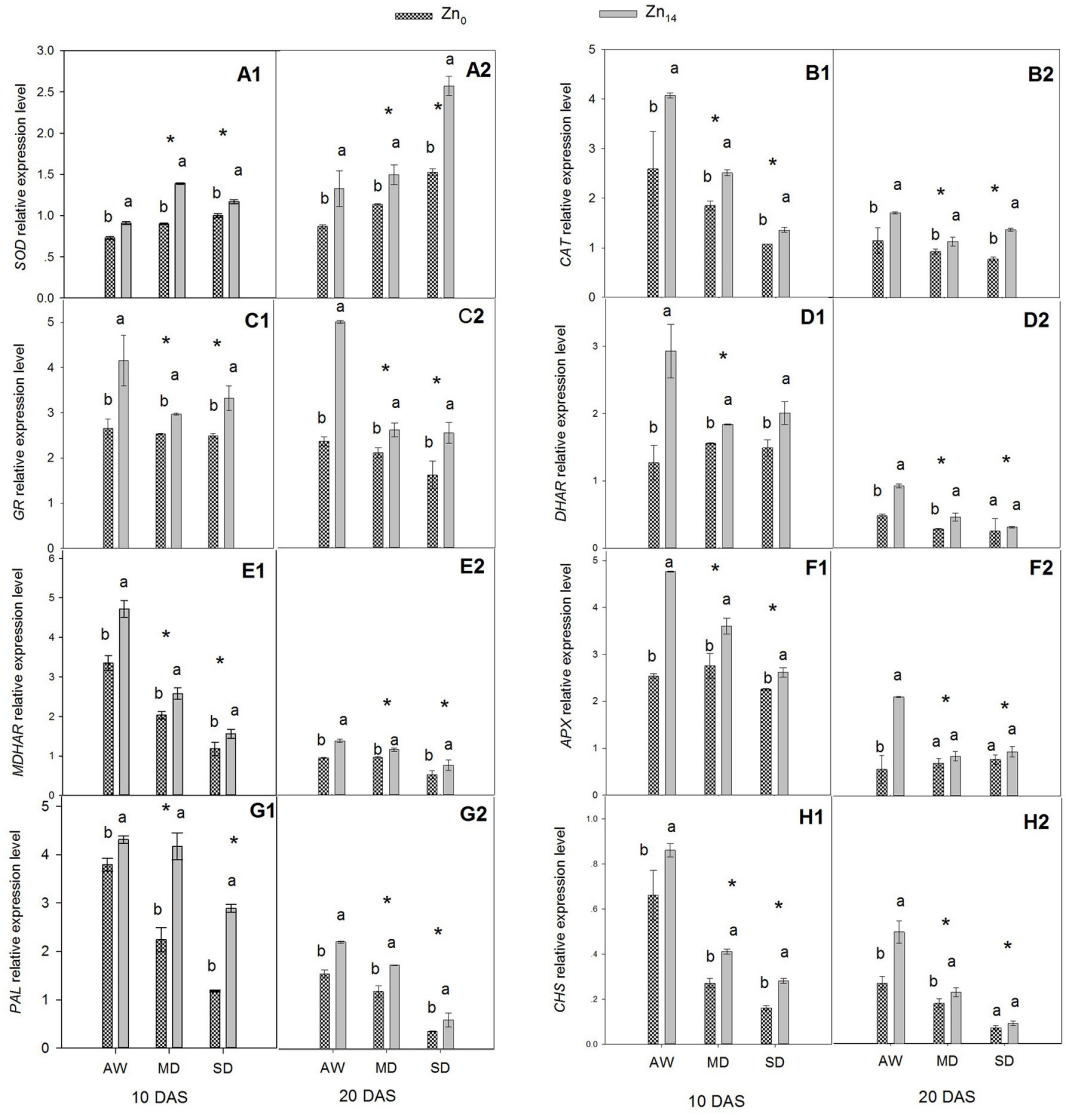
**Effect of Zn a plication on relative expression level of genes involved in antioxidant defense in flag leaves under different water conditions**. Vertical bars indicate the standard deviation of the mean; DAS, days after initiation of stress conditions; AW, adequate water; MD, moderate drought; SO, severe drought; Different lower case letters above the columns in the same test period indicate significant different by the LSD method (*p* < 0.05); ^*^above the columns at MD and SD indicate significant difference between drought stress and AW. **(A1, B1, C1, D1, E1, F1, G1, H1)** SOD, CAT, GR, DHAR, MDHAR, APX, PAL and CHS at 10 DAS, respectively. **(A2, B2, C2, D2, E2, F2, G2, H2)** SOD, CAT, GR, DHAR, MDHAR, APX, PAL and CHS at 20 DAS, respectively.

The relative expression levels of two antioxidant enzyme genes (*SOD* and *CAT*), four ASC–GSH cycle genes (*GR, DAR, MDAR*, and *APX*), and two flavonoid biosynthesis pathway genes (*PAL* and *CHS*) were presented in Figure 3. W, Zn application, and its interaction were all significant for the expression level of *SOD* at 10 and 20 DAS (Figures 3A1,A2). This said, only W treatment and Zn application had a significant effect on *CAT* expression (Figures 3B1,B2). Relative expression level of *SOD* increased, and *CAT* expression levels decreased when the wheat was exposed to drought stress. Zn_14_ application significantly increased *SOD* gene expression regardless of water condition, compared with the corresponding Zn_0_-treated plants. The expression level of *SOD* in wheat plants treated with Zn_14_ application increased by 54.4% compared with those treated with Zn_0_ under MD treatment at 10 DAS, and was almost two-fold greater under SD treatment at 20 DAS. A similar trend was observed in the expression of *CAT* in response to drought stress and Zn application. Compared with AW treatment at 10 DAS, MD, and SD stress decreased the *CAT* expression level by an average of 34.5 and 44.0%, respectively. However, Zn_14_ application increased *CAT* expression level by an average of 44.0% at 10 DAS, compared with Zn_0_ treatment.

The similar expression pattern with Zn application under different water treatment was observed for four ASC–GSH cycle genes (Figures 3C1,C2,D1,D2,E1,E2,F1,F2). W treatment, Zn fertilizer application and its interaction were all significant for these genes. The expression level of *GR, DHAR, MDHAR*, and *APX* decreased when the plants were exposed to drought stress. Compared with the corresponding AW treatment, MD and SD stress decreased the mean relative expression level of *GR* by 19.12 and 14.71% at 10 DAS, and 35.9 and 43.4% at 20 DAS, respectively. Zn_14_ application significantly increased *GR* expression both at 10 and 20 DAS regardless of the soil water regime. However, the biggest increase between Zn_0_ and Zn_14_ treatment was observed under the AW condition; *GR* expression with Zn_14_ application was 1.6 and 2.5 times that of Zn_0_ treatment at 10 and 20 DAS, respectively (Figures 3C1,C2). Similarly, the relative gene expression levels of *DHAR, MDHAR*, and *APX* in Zn_14_-treated plants were significantly higher than in the Zn_0_-treated plants regardless of the water treatment (except for *DHAR* and *APX* at 20 DAS). Compared with the corresponding Zn_0_ treatment at 10 DAS, Zn_14_ application increased the expression level of *DHAR* by 130.7, 17.9, and 18.9% under AW, MD, and SD treatment, respectively (Figure 3D1).

W treatment, Zn fertilizer application, and W × Zn interaction significantly affected the expression levels of *PAL* and *CHS* (Figures 3G1,G2,H1,H2). Drought stress significantly decreased the relative expression level of *PAL* and *CHS* at 10 and 20 DAS, compared with the well-watered treatment. However, Zn_14_ application significantly increased *PAL* and *CHS* expression regardless of water regime, compared with Zn_0_ treatment. At 10 DAS, the relative expression level of *PAL* with Zn_14_ application increased by 144.9% compared with Zn_0_ treatment under SD stress, and was almost two-fold greater under the MD treatment. Furthermore, a similar trend was observed for *CHS* expression under drought conditions with Zn application.

### Effect of Zn application on wheat grain yield and grain Zn concentration

As shown in Table 3 (pot experiment I), both W treatment and Zn application contributed significantly to the variation in number of grain per spike, yield and grain Zn concentration. Additionally, there was a significant W × Zn interaction on the number of grain per spike and grain Zn concentration. Compared with AW treatment, MD stress decreased yield by 15.1%, yet increased grain Zn concentration by 5.2%. Zn application significantly increased the number of grain per spike and grain yield whether under AW supply or MD treatment. Compared with Zn_0_ treatment, Zn_14_ application increased grain yield by an average of 15.4%. Zn_14_ increased the number of grain per spike by 3.5%, and 5.2% under AW and MD treatment, respectively, compared with the corresponding Zn_0_ treatment. Correspondingly, grain Zn concentration increased by 17.4 and 24.7% under AW and MD treatment, respectively. A similar trend was observed in grain yield and grain nutrient element concentration with soil water and Zn fertilizer treatment in pot experiment II (Table 3). TKW, yield, and grain Zn concentration were all significantly influenced by W and Zn treatment. W × Zn interaction had a significant effect on grain Zn concentration. Compared with the corresponding Zn_0_ treatment, Zn_14_ increased TKW by an average of 0.88 g. Zn application significantly increased grain yield and grain Zn concentration whether under AW or drought treatment. Compared with the corresponding Zn_0_ treatment, Zn_14_ increased the yield by 10.5, 22.6, and 28.2% under AW, MD, and SD treatment, respectively. Correspondingly, Zn_14_ increased the grain Zn concentration by 15.8, 9.7, and 32.8% under AW, MD, and SD treatment, respectively.

**Table 3:** **Effect of Zn fertilizer application on wheat yield, yield components and grain Zn concentration under different water conditions^a^^,^^b^^,^^c^^,^^d^^,^^e^**.

**Treatments**		**No. of spikes per pot**	**No. of grain per spike**	**TKW (g)**	**Yield (g.pot^−1^)**	**Grain Zn concentration (mg.kg^−1^)**
**POT EXPERIMENT I**						
AW	Zn_0_	30.5 ± 2.1a	35.60 ± 0.28b	45.30 ± 0.85a	40.31 ± 0.22b	35.44 ± 0.79b
	Zn_14_	32.5 ± 0.7a	36.85 ± 0.07a	46.26 ± 0.37a	46.10 ± 0.88a	41.61 ± 0.57a
	Mean	31.5A	36.22A	45.78A	43.21A	38.53B
MD	Zn_0_	29.2 ± 1.4a	33.75 ± 0.35b	43.40 ± 1.70a	33.86 ± 1.04b	36.08 ± 2.01b
	Zn_14_	30.2 ± 0.7a	35.50 ± 0.01a	44.40 ± 0.85a	39.49 ± 0.39a	45.00 ± 1.06a
	Mean	29.7A	34.63B	43.90B	36.68B	40.54A
	W	ns	^**^	^**^	^**^	^**^
	Zn	ns	^*^	ns	^**^	^**^
	W × Zn	ns	^*^	ns	ns	^**^
**POT EXPERIMENT II**						
AW	Zn_0_	34.0 ± 1.5a	36.41 ± 1.46a	42.28 ± 0.14b	43.25 ± 0.53b	34.38 ± 0.92b
	Zn_14_	34.2 ± 0.1a	37.21 ± 0.45a	43.10 ± 0.16a	47.80 ± 0.30a	39.82 ± 0.62a
	Mean	34.1A	36.81A	42.70A	45.53A	37.10B
MD	Zn_0_	32.1 ± 1.6a	35.06 ± 0.63a	40.87 ± 0.06a	31.80 ± 0.38b	37.04 ± 0.58b
	Zn_14_	31.3 ± 0.1a	36.63 ± 0.49a	41.41 ± 0.06a	38.98 ± 0.06a	40.64 ± 1.69a
	Mean	32.2A	35.87A	41.14B	35.42B	38.84B
SD	Zn_0_	31.2 ± 1.5a	32.99 ± 0.30a	38.48 ± 0.28b	23.13 ± 0.62b	34.31 ± 2.10b
	Zn_14_	32.2 ± 0.5a	33.22 ± 0.18a	39.75 ± 0.11a	29.66 ± 0.66a	49.57 ± 0.69a
	Mean	31.7A	33.11B	39.11C	26.39C	41.94A
	W	ns	^*^	^**^	^**^	^**^
	Zn	ns	ns	^**^	^**^	^**^
	W × Zn	ns	ns	ns	ns	^*^
**Field experiment**		**No. of spikes (**×**10**^4^**.ha**^−2^**)**	**No. of grain per spike**	**TKW (g)**	**Yield (kg.ha**^−2^**)**	**Grain Zn concentration (mg.kg**^−1^**)**
AW	Zn_0_	562.5 ± 95.5a	28.7 ± 1.6a	53.6 ± 0.14b	8204.0 ± 147.6b	35.06 ± 3.50b
	Zn_0.4_	632.5 ± 15.9a	30.0 ± 0.9a	54.0 ± 0.07a	9376.0 ± 110.5a	41.88 ± 1.25a
	Mean	597.5A	29.4A	53.8A	8790.0A	38.47A
RF	Zn_0_	577.5 ± 31.8a	23.4 ± 1.1a	51.4 ± 0.14b	6800.2 ± 243.2b	32.36 ± 0.47b
	Zn_0.4_	622.5 ± 31.8a	25.4 ± 0.7a	53.6 ± 0.07a	7984.8 ± 176.7a	43.97 ± 1.67a
	Mean	600A	24.4B	52.5B	7932.4B	38.17A
	W	ns	^**^	^**^	^**^	ns
	Zn	ns	ns	^**^	^**^	^**^
	W × Zn	ns	ns	^**^	^*^	^*^

a *Values expressed as mean ± standard deviation*.

b *Means in the same column within an irrigation regime followed by different lowercase letters are significantly different (p < 0.05)*.

c *Within a column mean, values for irrigation regimes followed by different uppercase letters are significantly different (p < 0.05)*.

d *W and Zn stand for water conditions and Zn fertilizer treatment, respectively*.

e* and ** *stand for significant different at p < 0.05 and p < 0.01), respectively; ns stand for no significant different*.

In the field experiment, irrigation, Zn fertilizer and W × Zn had a significant effect on TKW and grain yield, while Zn fertilizer and W × Zn had a significant effect on grain Zn concentration. RF treatment significantly decreased the number of grain per spike compared with the well-watered condition. However, foliar Zn application significantly increased TKW and grain yield. Compared with Zn_0_ application, foliar application of Zn increased grain yield and grain Zn concentration by 14.3 and 19.4%, 17.4 and 35.8%, under AW condition and RF treatment, respectively.

## Discussion

Zn is an essential plant nutrient and plays an important role in plant growth. It was reported that Zn application under adverse conditions, such as salt stress and cadmium toxicity, could increase chlorophyll a and b content and photosynthesis rate, and improve plant growth (Hassan et al., 2005; Tavallali et al., 2010). In this work, soil and foliar application of Zn increased SPAD and *Fv/Fm* improving plant photosynthetic characteristics under water stress. Similar findings have been reported in wild emmer wheat and chickpea where the application of Zn to the soil improved drought tolerance (Khan et al., 2003; Peleg et al., 2008). Moreover, foliar application of Zn increased photosynthesis rate and SPAD value in wheat leaves under drought stress (Karim et al., 2012). It has been hypothesized that chlorophyll synthesis is improved by Zn, which acts as a structural and catalytic component of proteins, enzymes, and as co-factor for normal development of pigment biosynthesis (Balashouri, 1995). Additionally, Zn is known to have a stabilizing and protective effect on biomembranes; improving the integrity of biomembranes may improve photosynthesis under stress (Cakmak, 2000).

Some low molecular weight antioxidant active substances, such as ASC, GSH, and phenolics, could increase resistance to drought (Rice-Evans et al., 1997; Chen et al., 2003). Cakmak (2000) suggested that Zn plays a protective role in preventing photoxidative damage catalyzed by ROS in chloroplasts. In this study, Zn application increased ASC and GSH content in wheat flag leaves under drought. An increase in ASC and GSH following application of Zn and boron was found in tobacco (Jiang et al., 2000). TPC and TFC are secondary metabolites which defend cells against ROS attack. We found that soil Zn application could improve TPC and TFC in wheat flag leaves under drought stress. Similarly, Zn treatments enhanced the accumulation of total phenols and flavonoids in berry (Song et al., 2015). Tavallali et al. (2010) found that soil application of Zn caused a significant increase in phenolics and ASC content in the leaves of pistachio, as compared with salt stress alone. The increase in antioxidants was mainly ascribed to Zn improving antioxidant biosynthesis. Zn possibly modulates GSH levels by regulating its biosynthesis or by protecting the reactive cysteine residue of GSH or through the efficient functioning of related enzymes (Foyer and Halliwell, 1976). These finding indicated that Zn application could increase antioxidant content to resist ROS damage induced by drought, which agreed with previous suggestions that micronutrient might greatly contribute to drought-stress tolerance by protection against oxidative damage of membranes (Cakmak, 2000; Ducic and Polle, 2005).

Zn is an essential plant nutrient and required as a metal component, or as a functional, structural, or regulator cofactor of many enzymes (Marschner, 1995). It is well-known that antioxidant enzymes, SOD, and CAT, constitute the first line of defense against ROS within a cell (Alscher et al., 2002). Wang and Jin (2007) found that Zn application significantly increased SOD activity in well-watered maize leaves, but no significant difference was observed between Zn treated-plant and non-Zn treatment under drought. In our experiments, Zn soil application was found to increase transcription of *SOD* in wheat flag leaves regardless of the soil water regime. The different responses of SOD activity and *SOD* gene expression in response to Zn application was mainly attributed to the different growth stages and the amount of Zn supplied. In a previous study, Zn fertilizer was applied at a rate of 5.0 mg kg^−1^ soil to fully expanded young maize leaves 35 days after planting. Here, the Zn concentration was 14.0 mg kg^−1^ soil and wheat flag leaves were used for gene expression analysis. Gao et al. (2009) found that both *SOD* gene expression and the activity of SOD in cucumber seedling leaves were induced by increasing Zn^2+^ under low temperature stress. Therefore, increasing Zn^2+^ could increase the capacity of scavenging ROS by inducing gene expressions of *SOD* and elevating activities of SOD. Our experiment did not test SOD activity in wheat flag leaves following Zn fertilizer application however, the enhanced *SOD* expression levels suggested that Zn application could improve drought resistance of wheat plants. Similar results were also reported by Yavas and Unay (2016); the activity of SOD and CAT increased significantly in drought + Zn wheat in comparison with drought stress.

ASC–GSH cycle and phenolic biosynthesis are involved in the non-enzymatic anti-oxidative system. The enzymes involved in the ASC–GSH cycle include MDHAR, DHAR, GS, and APX. Liu et al. (2012) suggested that APX and MDAR may play more important roles in stress tolerance in plants. In our experiments, Zn application was found to increase transcription of *MDHAR, DHAR, GS*, and *APX* in plants under drought stress. The increased activity of APX following Zn supply was found in pistachio under salt stress (Tavallali et al., 2010). PAL and CHS are two important enzymes in the initial stages of the flavonoid biosynthesis pathway. It was reported that exogenous silicon significantly increased the transcript abundance of *PAL* and *CHS* in wheat and rice (Fleck et al., 2011; Ma et al., 2016). Our results showed that the expression level of *PAL* and *CHS* was elevated in Zn-treated wheat leaves, as compared with Zn_0_ treatment. Zn has been suggested to alter gene expression by occupying a site on a transcription factor which increases the rate of transcription (Cousins, 1998). These results suggest that Zn application could improve the transcription level of genes involved in plant antioxidative defense, thus enhancing the resistance of wheat to drought stress.

Zn fertilizer application has been recommended as an effective way to increase grain yield and Zn concentration. However, soil moisture plays a major role in providing micronutrients to plant roots. Wang and Jin (2007) suggested that Zn fertilizer was recommended for maize plants and supplied with ample water. In our experiments, application of Zn increased grain yield, and grain Zn concentration whether in AW supply or drought stress, as compared with non-Zn treated plants. A similar result was reported by Karim et al. (2012), who found that foliar application of Zn increased wheat grain yield as well as raising grain Zn concentration under drought. The increasing grain yield and grain Zn concentration was due to the plant obtaining more Zn through the soil or leaf. Interestingly, we found that different soil water conditions with Zn_0_ application did not significantly influence grain Zn concentration, while plants treated with Zn_14_ application under SD treatment had the highest Zn concentration. One potential explanation is that drought stress was imposed from the heading stage; plants may take sufficient Zn in the early stages under good experimental conditions. Alternatively, sufficiently high Zn is needed to alleviate drought stress by contributing to detoxification of ROS (Carvalho, 2008).

Application of Zn fertilizer could increase grain yield and grain Zn concentration regardless of soil water supply conditions. Moreover, Zn fertilizer application had more effect on improving grain Zn concentration under SD condition, as compared with AW supply. Zn application could alleviate the oxidative stress of wheat through transcriptional regulation of multiple defense pathways, such as antioxidant enzymes, the ASC–GSH cycle, and flavonoid secondary metabolism. Zn application may improve the effects caused by drought stress through enhancing reactive species biosynthesis. This study provides useful information for underlying physiological and biochemical mechanisms involving Zn application and plant tolerance to stress. Furthermore, this study provides evidence for the use of Zn application in arid and semiarid environments to increase grain yield and Zn concentration.

## Author contributions

The work presented here was carried out in collaboration between all authors. CW and TG defined the research theme and co-designed experiments, and discussed analyses. DM designed the methods, experiments and wrote the manuscript. DS carried out the experiments and analyzed the data. YX co-worked on associated data collection and their interpretation. HQ and HD co-worked on laboratory analysis. JH and XH contributed the Zn element analysis. All authors have contributed to, seen and approved the manuscript.

### Conflict of interest statement

The authors declare that the research was conducted in the absence of any commercial or financial relationships that could be construed as a potential conflict of interest.
